# Preoperative Embolization in Surgical Resection of Cervical Paragangliomas: Usefulness and Current Evidence

**DOI:** 10.7759/cureus.48278

**Published:** 2023-11-04

**Authors:** Sunil Rajendran, IP Yadev, Ramachandran Sreekumar

**Affiliations:** 1 Vascular Surgery, Starcare Hospital, Kozhikode, IND; 2 General Surgery, Government Medical College, Thiruvananthapuram, Thiruvananthapuram, IND; 3 General Surgery, Government Medical College, Thiruvanthapuram, Thiruvananthapuram, IND; 4 Vascular Surgery, Sree Chitra Tirunal Institute for Medical Sciences and Technology, Thiruvananthapuram, IND

**Keywords:** surgical excision of tumour, cranial nerve palsy, preoperative embolisation, carotid body tumour, cervical paraganglioma

## Abstract

Background: Cervical paragangliomas (CPG) are slow-growing tumours, most of which are highly vascular, making surgical resection challenging. Preoperative embolisation of afferent arteries has been recommended to facilitate operative dissection, reduce perioperative blood loss, and shorten the duration of the operation. However, there is conflicting evidence on the benefits of preoperative embolisation on surgical outcomes, operative time, and bleeding.

Objectives: The objective of this study was to compare the perioperative parameters and outcomes like duration of surgery, blood transfusion, duration of stay in the ICU, cranial nerve injuries, and postoperative stroke between patients who underwent excision of CPGs with and without preoperative embolisation.

Methods: This is a retrospective study conducted at the Division of Vascular Surgery, Sree Chitra Tirunal Institute of Medical Sciences and Technology, Thiruvananthapuram, Kerala, India. The study included a review of the medical records of 32 patients who underwent excision of CPGs. Patients who underwent surgical resection without preoperative embolisation (SR) were compared with those who underwent surgical resection with embolisation (SREMB). Statistical analysis was done in R statistical software (R Foundation for Statistical Computing, Vienna, Austria). Categorical variables were reported in absolute numbers and percentages continuous variables were compared with an unpaired Mann-Whitney U test. The chi-square test was used to compare the categorical data.

Results: Out of 32 patients included in this study, 13 (40.6%) patients were included in the SREMB group. Between the two groups, there were no significant differences in operative time (120 vs. 150 minutes; p = 0.59), blood transfusion requirement (0.69 vs. 0.37 units; p = 0.39) and ICU stays (1 vs. 1; p = 0.56). Postoperative cranial nerve injury was significantly more in the SREMB group (6 vs 1; p = 0.01) but stroke (0 vs 2; p = 0.50) was not statistically significant between the two groups.

Conclusion: In this study, we found that there was insufficient evidence to support routine preoperative embolisation in CPG. Hence, preoperative embolisation should only be used in a very select group of patients.

## Introduction

Cervical paragangliomas (CPGs) are slow-growing tumours of neural crest origin commonly arising from the carotid body, vagal nerve, or jugular bulb. Among these, carotid body tumours (CBT) are the most common, while others are relatively rare. These tumours have malignant potential in over 10-20% of cases and surgical resection is the treatment of choice [1,2]. However, it may be complicated by excessive haemorrhage, stroke, and cranial nerve injuries (CNIs).

The relationship of these tumours with adjoining external and internal carotid arteries forms the basis of the Shamblin classification, which classifies CBT into three grades [3]. Higher Shamblin tumour grades have been reported to have higher operative complications and morbidity [4-6]. CPG predominantly receives its blood supply from the branches of the external carotid artery (ECA) [7].

Preoperative embolisation of afferent arteries has been recommended to facilitate operative dissection, reduce perioperative blood loss, and shorten the duration of the operation [6,8]. However, there is conflicting evidence on the benefits of preoperative embolisation on surgical outcomes, operative time, and bleeding [4,7,9-13]. Embolization may result in increased morbidity due to complications like stroke, carotid sinus hypersensitivity, and inadvertent embolisation of other vessels [14-16]. In this context, we compared the duration of surgery, blood loss, CNI, and the surgical outcome between surgical excision of CPGs with and without preoperative embolisation.

## Materials and methods

Our institutional practices have evolved and preference for pre-operative embolisation has markedly decreased over the years. This was a retrospective audit of 10-year data, of the medical records of patients who had undergone surgery for CPGs at the Division of Vascular Surgery at Sree Chitra Tirunal Institute for Medical Sciences and Technology (SCTIMST), Thiruvananthapuram, Kerala, India. All patients included in this study were referred to SCTIMST from various centres with clinical features suggestive of a CPG. The initial diagnosis was made clinically and by ultrasound of the neck followed by advanced imaging studies like CT scan, MRI, and digital subtraction angiography (DSA).

The decision regarding the need for embolisation was largely based on the discretion of two senior surgeons with experience of more than 15 years, in consultation with the interventional radiologists. In all patients who underwent preoperative embolisation, except one, the procedure was done a maximum of three days, that is zero to three days, prior to surgical resection. The embolisation procedure was done with particles like Gelfoam (GF), hydrogel (HG), or Polyvinyl Alcohol (PVA), or with direct injection of bucrylate into the tumour. We collected the data from the electronic case records and the patients were followed up in our clinic.

Patients were divided into two groups: (i) Those who underwent surgical resection without embolisation (SR) and (ii) those who underwent surgical resection after embolisation (SREMB). Basic demographic features like age, gender, laterality, duration of symptoms, presence of diabetes, hypertension, and Shamblin types were compared between the two groups. We also analysed factors like duration of surgery, need for blood transfusion, duration of stay in the ICU, CNI, and postoperative stroke between two groups of patients. 

A sub-group analysis between SR and SREMB groups was also done for patients with tumour size (≥5 cm) and those with Shamblin 2 and 3 tumours. The above criteria were used as patients with tumours of larger size and higher Shamblin grades are usually considered candidates for preoperative embolization. 

Normally distributed variables were expressed as mean, standard deviation, and non-normal with median and interquartile range. We reported categorical variables in absolute numbers and percentages. We compared continuous variables between the two groups with an unpaired Mann-Whitney U test. The chi-square test was used to compare the categorical data. All statistical analysis was done in R statistical software (R Foundation for Statistical Computing, Vienna, Austria) [17].

## Results

There were 32 CPG patients, with one patient having bilateral tumours, operated on within a one-year duration. Of these, 27 (84.4%) were CBTs, four (12.5%) were glomus vagale tumours, and one (3.1%) was a glomus jugulare tumour. The median age was 35.3 (interquartile range (IQR) 24-46), there were 12 (41%) females and 19 (59%) males and the average duration of the presentation was 15 (IQR 6-30). The tumour was on the right side in 16 (52%) and on the left side in 15 (48%) and one patient, as mentioned, had bilateral involvement. Most of the patients were normotensive (97%) and non-diabetic (94%) and only two patients were smokers. At presentation, one patient had a history of transient ischemic attack and preoperative cranial nerve palsies with involvement of 9th, 10th and 12th nerves were present in three patients. 

Of the tumours, 50% were Shamblin type 2 followed by type 1 (28.1%) and type 3 (21.9%). The median operating time was 128 minutes (IQR 90-212). Most of the patients (72%) did not require any blood transfusion with five patients transfused with one unit of blood and three patients with three units of blood. Complete tumour excision was done in all cases. The mean duration of ICU stay was 2.1 (2.3) days with an average total duration of hospital stay of 6.2 (2.9) days. Two patients had a postoperative stroke and seven patients (21.9%) had postoperative cranial nerve palsy. The nerves involved in postoperative cranial nerve palsy were 9th, 10th, 12th, and recurrent laryngeal nerves. Presentations included hoarseness of voice and Horner's syndrome (Table 1). Two patients had Horner's syndrome, one each in the SR and SREMB groups. Out of 32 patients, 13 patients underwent preoperative embolisation and baseline characteristics were comparable between the SR group (n=19) and SREMB groups (n=13) (Table 1). 

**Table 1:** Comparison of baseline characteristics Data presented as median (interquartile range) or number (%). SREMB, Surgical resection after embolisation; SR, Surgical resection without embolisation

	Total	SREMB	SR	p-value
	(n=32)	(n=13)	(n=19)	
Age	35.3 (14.4)	36.6 (15.9)	34.5 (13.6)	0.70
Sex: Male	19 (59.4%)	9 (69.2%)	10 (52.6%)	0.57
Duration (months)	15 (5.50-25.5)	8 (3-18)	24 (6.50-35)	0.07
Hypertension	1 (3.12%)	1 (7.69%)	0 (0.00%)	0.41
Diabetes Mellitus	2 (6.25%)	1 (7.69%)	1 (5.26%)	1.00
Smoker	2 (6.25%)	1 (7.69%)	1 (5.26%)	1.00
Stroke	1 (3.12%)	1 (7.69%)	0 (0.00%)	0.41
Cranial nerve palsy	3 (9.38%)	1 (7.69%)	2 (10.5%)	1.00
Shamblin type:				0.52
1	9 (28.1%)	5 (38.5%)	4 (21.1%)	
2	16 (50.0%)	5 (38.5%)	11 (57.9%)	
3	7 (21.9%)	3 (23.1%)	4 (21.1%)	

**Table 2:** Comparison of perioperative outcomes between SREMB and SR groups. Data presented as median (interquartile range) or number (%). SREMB, Surgical resection after embolisation; SR, Surgical resection without embolisation; ICU, Intensive care units; CNI, Cranial nerve injury

	Total	SREMB	SR	p-value
	(n=32)	(n=13)	(n=19)	
Operating time (minutes)	128 (90-212)	150 (90-160)	120 (90-240)	0.59
Days in ICU	1 (1-2)	1 (1-2)	1 (1-2)	0.56
Postoperative stroke	2 (6.25%)	0 (0.00%)	2 (10.5%)	0.50
Postoperative CNI	7 (21.9%)	6 (46.2%)	1 (5.26%)	0.01
Blood Transfusion (units)	0.50 (0.95%)	0.69 (1.18%)	0.37 (0.76%)	0.39

A comparison of various outcomes between the SR group and SREMB groups showed no statistically significant difference in the operating time, the requirement of blood transfusion, total hospital stay, days in ICU, or postoperative complications like postoperative stroke. However, postoperative cranial nerve palsy was significantly more in the SREMB group (Table 2). When surgical outcomes of patients with Shamblin type 2 and 3 tumours were analysed with respect to those who underwent SREMB (n=8) vs. those who underwent SR (n=15), cranial nerve palsies were more in the SREMB group (62.5% vs. 6.67%, p=0.01). Other parameters like operating time, ICU stay, postoperative stroke, and blood transfusions were almost similar in both groups (Table 3).

**Table 3:** Comparison of perioperative outcomes between SREMB and SR groups in patients with Shamblin 2 and 3 tumours. Data presented as median (interquartile range) or number (%). SREMB, Surgical resection after embolisation; SR, Surgical resection without embolisation; ICU, Intensive care units; CNI, Cranial nerve injury

	Total	SREMB	SR	p-value
	(n=23)	(n=8)	(n=15)	
Operating time (minutes)	150 (90-240)	150 (135-165)	135 (90-248)	1.00
Days in ICU	2 (1-2)	1.50 (1-2)	2 (1-2.50)	0.48
Postoperative stroke	2 (8.70%)	0 (0.00%)	2 (13.3%)	0.53
Postoperative CNI	6 (26.1%)	5 (62.5%)	1 (6.67%)	0.01
Blood transfusion (units)	0.22 (0.52)	0.25 (0.71)	0.20 (0.41)	0.86

Finally when patients with large tumours (≥5 cms) who underwent SREMB (n=10) were compared to patients with large tumours in the SR group (n=8), cranial nerve palsies were seen to be more in the SREMB group (50% vs. 12.5%, p=0.15). However, this was not statistically significant. There were also non-significant differences in operating time, ICU stay, postoperative stroke, and blood transfusions in the SREMB group compared to the SR group (Table 4).

**Table 4:** Comparison of perioperative outcomes between SREMB and SR groups in patients with tumours more than 5 cm in size. Data presented as median (interquartile range) or number (%). SREMB, Surgical resection after embolisation; SR, Surgical resection without embolisation; ICU, Intensive care units; CNI, Cranial nerve injury

	Total	SREMB	SR	p-value
	(n=18)	(n=10)	(n=8)	
Operating time (minutes)	142 (90-202)	150 (97.5-175)	112 (90-240)	0.93
Days in ICU	2 (1-2)	1.50 (1-2)	2 (1-2)	0.45
Postoperative stroke	1 (5.56%)	0 (0.00%)	1 (12.5%)	0.44
Postoperative CNI	6 (33.3%)	5 (50.0%)	1 (12.5%)	0.15
Blood transfusion (units)	0.50 (0.99)	0.70 (1.25)	0.25 (0.46)	0.31

## Discussion

In this study, various outcomes between SR and SREMB were compared and the authors found that there was insufficient evidence to say there were differences in the outcomes like operative time, blood transfusion, days in ICU, or postoperative stroke between the two groups.

This study showed a slightly longer, though not statistically significant operating time, for resection in the SREMB group, similar to observations made in a few studies, whereas other studies reported higher time with the non-embolization group [8,9,18]. This strengthens our view and experience that there is not much evidence in favour of preoperative embolization. Our result is consistent with the metanalysis by Abu-Ghanem et al. [13]. A possible reason for the similar operative time between the two groups may be due to a selection bias wherein larger tumours may have been selected preferentially for preoperative embolization. Moreover, the presence of peritumoral inflammation subsequent to embolisation as reported by a few authors might explain the slightly increased duration of surgery in the SREMB group [9]. However, all these studies are observational studies with inherent selection bias, which could explain the contrasting evidence, in addition to the explanations given.

In the current study, a comparison of the total duration of stay in the ICU failed to show any significant difference, though the surgery-alone group spent more days in intensive care. However, this was clinically and statistically not significant. The total duration of stay in the ICU was longer in the present study, compared to some studies in the literature [19]. The duration of ICU stays need not show the severity of the conditions. It could depend on the institutional practices and doctor's preferences. 

There were two postoperative strokes in the SR group, whereas no stroke occurred in the SREMB group, but this was not statistically significant. Stroke in one patient was subsequent to the surgical procedure warranting ligation of the internal carotid artery and in another due to thrombosis of the artery. Our results were comparable with other studies, in which cases there were instances of preoperative ligation of internal carotid arteries [10,20]. Few studies reported less incidence of postoperative strokes in patients undergoing embolization prior to surgical resection whereas, as in the current study, Texakalidis et al. reported similar stroke rates in two groups [6,21,22]. 

In this series, more CNI were observed in the SREMB (n=6) when compared to the SR group (n=1), p=0.01. There was a significant difference in the occurrence of CNI between the two groups similar to other studies [6,9]. Osofsky et al. reported an increased incidence of CNI in the embolized groups and authors attributed this to the peritumoral inflammation resulting in poorly defined planes of dissection at the surgery. 

Similar to other studies the requirement of blood transfusion or blood loss also did not seem to be different between the two groups [9,23]. 

Increased tumour size and Shamblin grades appear to increase the difficulty of surgery and are associated with increased complications [4,5,9]. However, like in the current study, there seems to be no convincing evidence to support preoperative embolization in tumours more than 5 cm in size and in those with Shamblin grades 2 and 3. These observations were in line with those reported in the study by Osofsky et al., in which the authors reported increased operative time and inferior surgical outcomes in the SREMB group [9].

Our study has many limitations. One limitation of this study is its retrospective design, which is subject to various forms of bias, such as selection bias and recall bias. Selection bias may occur if the decision to perform preoperative embolization was based on certain patient or tumour characteristics, which could influence the results. Recall bias may occur if patients or physicians recall information inaccurately. Another limitation of this study is the low sample size to power all the hypotheses. However, CPGs are rare and the vascular unit involved in this study caters to a wide geographical area. Another limitation of our study is the potential selection bias. Being a referral centre with vascular specialisation, there was a possibility that only complicated cases and higher Shamblin types were preferentially referred. It could have affected the results. 

## Conclusions

There was insufficient evidence to support routine preoperative embolisation in CPG. Apart from inherent complications of embolisation, there seems to be a slightly increased incidence of intraoperative CNI in this group of patients. The results of this study have important implications for the management of cervical paragangliomas. It suggests that preoperative embolization may not be necessary for all patients with CPG and that it may not provide significant benefits in terms of surgical outcomes, blood loss, and postoperative complications. This could have important cost-saving implications for healthcare systems, as embolization procedures can be expensive and resource-intensive. Despite the limitations discussed, the study provides valuable insights into the safety and efficacy of preoperative embolization for CPGs, and the results should be interpreted with caution and further large-scale studies should be conducted to confirm the findings.
